# Faraday Waves-Based Integrated Ultrasonic Micro-Droplet Generator and Applications

**DOI:** 10.3390/mi8020056

**Published:** 2017-02-14

**Authors:** Chen S. Tsai, Rong W. Mao, Shirley C. Tsai, Kaveh Shahverdi, Yun Zhu, Shih K. Lin, Yu-Hsiang Hsu, Gerry Boss, Matt Brenner, Sari Mahon, Gerald C. Smaldone

**Affiliations:** 1 Department of Electrical Engineering and Computer Science, University of California, Irvine, CA 92697, USA; rwmao@uci.edu (R.W.M.); kshahver@uci.edu (K.S.); yunz@uci.edu (Y.Z.); shihkail@uci.edu (S.K.L.); 2 Department of Chemical Engineering and Materials Science, University of California, Irvine, CA 92697, USA; sctsai@csulb.edu; 3 Institute of Applied Mechanics, National Taiwan University, Taipei 106, Taiwan; yhhsu@mail.iam.ntu.edu.tw; 4 School of Medicine, University of Calfironia, San Diego, La Jolla, CA 92093, USA; gboss@ucsd.edu; 5 Division of Pulmonary and Critical Care Medicine, Beckman Laser Institute & Medical Clinics, School of Medicine, University of California, Irvine, CA 92697, USA; mbrenner@uci.edu (M.B.); mahonsb@uci.edu (S.M.); 6 Pulmonary, Critical Care and Sleep Medicine Division, State University of New York at Stony Brook, New York, NY 11790, USA; gerald.smaldone@stonybrook.edu

**Keywords:** Faraday waves, Faraday instability, multiple Fourier horns (MFH), MFH ultrasonic nozzle, ultrasonic micro droplet generator, integrated ultrasonic nebulizer, ultrasonic nebulizer, onset threshold for droplet ejection, clogging free, monodisperse, polydisperse, pulmonary (inhalation) drug delivery

## Abstract

An in-depth review on a new ultrasonic micro-droplet generator which utilizes megahertz (MHz) Faraday waves excited by silicon-based multiple Fourier horn ultrasonic nozzles (MFHUNs) and its potential applications is presented. The new droplet generator has demonstrated capability for producing micro droplets of controllable size and size distribution and desirable throughput at very low electrical drive power. For comparison, the serious deficiencies of current commercial droplet generators (nebulizers) and the other ultrasonic droplet generators explored in recent years are first discussed. The architecture, working principle, simulation, and design of the multiple Fourier horns (MFH) in resonance aimed at the amplified longitudinal vibration amplitude on the end face of nozzle tip, and the fabrication and characterization of the nozzles are then described in detail. Subsequently, a linear theory on the temporal instability of Faraday waves on a liquid layer resting on the planar end face of the MFHUN and the detailed experimental verifications are presented. The linear theory serves to elucidate the dynamics of droplet ejection from the free liquid surface and predict the vibration amplitude onset threshold for droplet ejection and the droplet diameters. A battery-run pocket-size clogging-free integrated micro droplet generator realized using the MFHUN is then described. The subsequent report on the successful nebulization of a variety of commercial pulmonary medicines against common diseases and on the experimental antidote solutions to cyanide poisoning using the new droplet generator serves to support its imminent application to inhalation drug delivery.

## 1. Introduction

**Figure 1:**
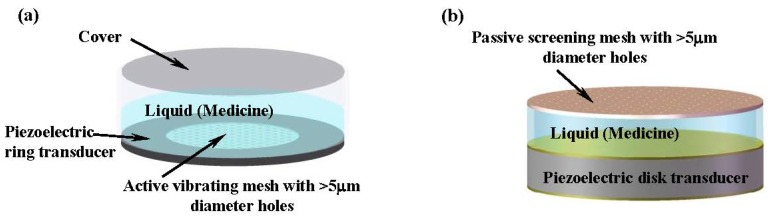
Basic architectures of advanced commercial ultrasonic nebulizers: (**a**) active vibrating mesh with ring transducer; (**b**) passive screening mesh with disk transducer.

Techniques for the generation of micro droplets have continued to be of great interest as the resultant droplet generators will facilitate various important applications. Current commercial ultrasonic droplet generators for inhalation drug delivery (nebulizers) utilize either a vibrating piezoelectric transducer together with an active vibrating mesh (Figure 1a) or a passive screening mesh (Figure 1b) [1,2] to produce micro droplets. However, since various undesirable mechanisms such as jetting, impinging, and cavitation, in addition to capillary wave mechanisms, are also associated with the droplet ejection, the size distributions of the droplets (aerosols) produced by such commercial droplet generators are very broad (polydisperse) and uncontrollable. Furthermore, although the mesh helps to select droplets of smaller diameters, it not only wastes expensive drugs but also causes clogging of the device by the viscous medicines. In addition to the Faraday waves-based multiple Fourier horn ultrasonic nozzles to be reviewed in detail in this paper, nozzleless droplet ejectors utilizing acoustic lens [3], liquid horn structure [4], PZT/tapered glass capillary [5], micro-machined transducers with annular piezoelectric disk [6,7,8], droplet generators using focused surface acoustic waves (SAW) [9], and planar SAW [10] were explored in recent years. Note that, since the basic ejector element of the first three droplet generators [3,4,5] produces only one droplet at a time, two-dimensional (2-D) array elements and electrical drivers are required to obtain desirable droplet throughput. The fourth droplet generator [6,7,8] suffers from the same deficiencies as the current commercial droplet generators. While the focused SAW-based droplet generator [9] suffers from low droplet throughput, the planar SAW-based droplet generator [10] has yet to demonstrate a capability for producing narrow and controllable droplet size distribution.

The new clogging-free micro-droplet generator presented here utilizes MHz Faraday waves excited by ultrasound with MHz silicon-based multiple Fourier horns (MFHs) in cascade and in resonance to generate micro droplets of controllable size and narrow size distribution. The enhanced vibration amplitude at the tip of the MFH ultrasonic nozzle (MFHUN) facilitates the onset threshold for Faraday wave formation, amplification, and subsequent ejection of such desirable droplets. 

**Figure 2:**
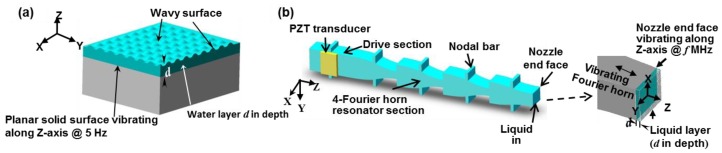
(**a**) Classical planar geometry for Faraday wave formation at low drive frequency; (**b**) 3-D architecture of MHz 4-Fourier horn ultrasonic nozzle depicting the geometry of its end face and the liquid layer.

Faraday waves were first observed as the wavy surface of a water layer resting on a planar elastic solid surface subjected to perpendicular mechanical vibration, as depicted in Figure 2a, at a very low drive (or forcing) frequency of 5 Hz by Faraday in 1831 [11] and were subsequently analyzed by Rayleigh (1883) and many others [12,13]. Faraday instability, the underlying physical mechanism for Faraday wave formation and amplification, has been studied extensively based on Faraday’s planar geometry since but mostly at low drive frequencies ranging from tens to thousands of Hz. See, for examples, the references cited in [14]. At such low drive frequencies, various standing-wave patterns were observed on the free liquid surface when the vibration amplitude (displacement) on the solid surface reached the onset threshold for Faraday wave formation. However, in the few reports on experiments at such low drive frequencies, droplet ejection was found to take place only when the vibration amplitude on the solid surface was much higher than the onset threshold for Faraday wave formation [15]. In stark contrast, our recent theoretical and experimental findings have shown that at the much higher drive frequency of MHz, the vibration amplitude onset threshold for Faraday wave formation is much lower, and the vibration amplitude required for subsequent droplet ejection is only slightly higher than the onset threshold for Faraday wave formation [16,17]. Accordingly, the enhanced vibration amplitude at the end face of the distal horn of the MHz multiple Fourier horns in resonance can readily facilitate the onset threshold for MHz Faraday wave formation and amplification and subsequent micro droplet ejection. 

## 2. Silicon-Based MHz Multiple Fourier Horn Ultrasonic Nozzles

### 2.1. Multiple Fourier Horn Nozzle Architecture and Working Principle

Silicon-based multiple Fourier horns (MFHs) in cascade and vibrating at a single resonance frequency at MHz, as shown in Figure 2b, are used to mimic Faraday’s classical low-frequency experiment (Figure 2a) at MHz drive frequency. The multiple Fourier horn ultrasonic nozzle (MFHUN) with its *Z*-axis along the <110> direction of the primary-flat silicon wafer is fabricated using the well-established micro-electromechanical system (MEMS) technology [18]. The ultrasonic nozzle consists of a drive section and a resonator section. A pair of identical lead zirconate titanate (PZT) piezoelectric plates are bonded on the top and bottom faces of the drive section to excite mechanical vibrations along the nozzle axis (*Z*-axis). The resonator section is made of multiple Fourier horns in cascade. Each basic horn is half a wavelength in length. The nozzle is designed to vibrate at a single resonance frequency *f* of the MFHs. Note that any unwanted modes such as non-longitudinal vibration modes (torsional mode, for example) are suppressed due to the limited bandwidth or half-power width (<2 kHz) of the designed resonance frequency of MHFs. The resultant vibration amplitude (displacement) on the nozzle tip (end face of the distal horn) is greatly enhanced due to resonance with a gain of *M^n^* for an *n*-Fourier horn nozzle in which *M* is the magnification of displacement for each basic horn; namely, the ratio of displacement on the horn end face (tip) to that on the horn base. For example, for a 3-Fourier horn nozzle with *M* = 2, the total gain in displacement, namely, the ratio of the displacement on the distal horn tip to that on the proximal horn base, will be 8 (= 2 × 2 × 2). The enhanced vibration amplitude readily facilitates the onset threshold required for micro-droplet ejection.

### 2.2. Simulation and Design

A three-dimensional (3-D) finite element method (FEM) simulation using the commercial ANSYS Program (ANSYS Inc., Canonsburg, PA, USA) is carried out first for vibration mode shape analysis and then for electrical impedance analysis [18,19]. The mode shape analysis determines the nozzle resonance frequency of the pure longitudinal vibration mode; the electrical impedance analysis determines the longitudinal vibration amplitude on the nozzle tip and the electrical impedance at the nozzle resonance frequency. Nozzles of various designs in terms of resonance frequency, the number of basic horns with various values for *M*, and horn length can be simulated accordingly prior to fabrication.

### 2.3. Fabrication and Characterization

**Figure 3:**
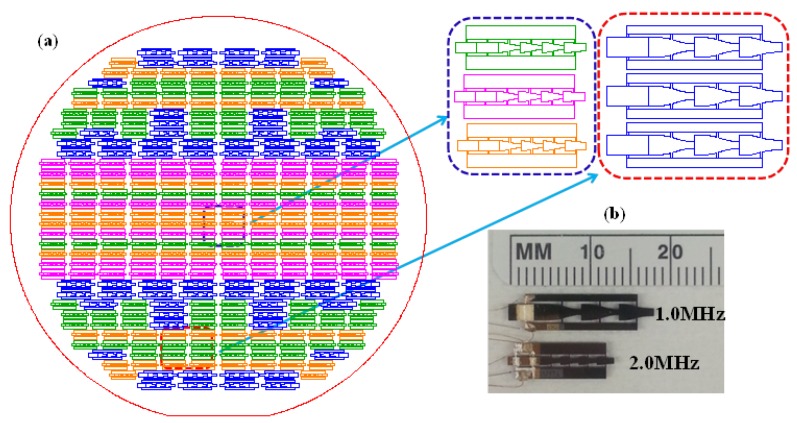
(**a**) Layout of nozzles in an 8-inch silicon wafer; (**b**) MEMS-fabricated 1.0 and 2.0 MHz nozzles.

The silicon-based MFHUNs of various designs, in terms of the aforementioned specifications, are then fabricated using MEMS technology [18,19]. The drive and resonator sections of the MFHUN are formed in a single-fabrication step using an inductive coupled plasma (ICP) process [20]. Note that the nozzles of identical or different design specifications can be fabricated simultaneously in a common silicon wafer. Figure 3a shows an example of the nozzles’ layout in an 8-inch wafer, widely used in the microelectronics industry. The individual nozzles are then diced using ICP; the drive section is bonded with a pair of identical PZT plates and connected with electrical wires. Examples of the resultant centimeter-size nozzles of 1.0 and 2.0 MHz drive frequencies are shown in Figure 3b. Clearly, a large number of nozzles of various designs can be batch-fabricated to drastically lower the unit cost of the nozzles.

**Figure 4:**
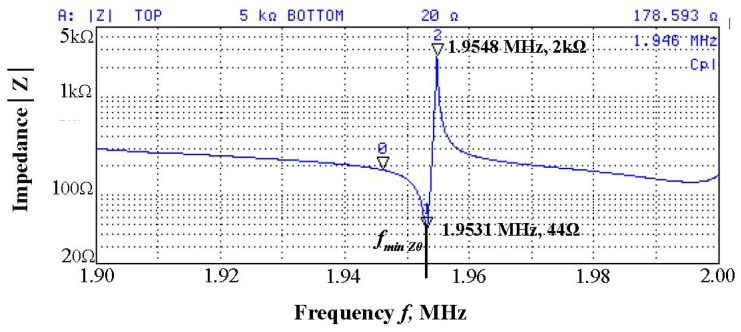
Typical impedance curve of 2 MHz 4-Fourier horn nozzles.

The only essential characterization for the fabricated nozzles prior to atomization (droplet ejection) experiments is to measure the impedance curve with and without a liquid layer on the end face of the nozzle tip using Agilent impedance analyzer model 4294A [19,21], from which the drive frequency and the electrical drive power are determined. A typical impedance curve for a 2 MHz 4-Fourier horn nozzle (Figure 4) in absence of a liquid layer shows clearly the resonance and anti-resonance frequencies (1.9531 and 1.9548 MHz) at minimum and maximum impedances of 44 Ω and 2 kΩ, respectively; the separation between these two frequencies (called half-power bandwidth, HW) is 1.7 ± 0.1 kHz. The curve clearly shows no other modes of vibration within the frequency range from 1.9 to 2.0 MHz because, as mentioned in Section 2.1, any unwanted modes are suppressed by the multiple Fourier horns in resonance. As a layer of liquid such as water was accumulated on the nozzle end face prior to atomization, the resonance frequency was lowered as a result of increased loading. For example, for a water layer 56 µm thick, the measured resonance frequency down shift was 7.1 kHz.

The simulated longitudinal vibration amplitude on the nozzle tip determined by the electrical impedance analysis (at electrode voltage of 1.0 V) is then used to determine the threshold voltage required to generate the onset threshold or critical vibration amplitude (*h*_cr_) for the Faraday wave formation and subsequent amplification to be presented in Section 3. Finally, the threshold voltage thus obtained, together with the resistive part of the impedance found, is then used to determine the electrical drive power required for droplet ejection. In short, the greatly enhanced peak vibration amplitude (*h*) on the nozzle end face of the MFHUN due to resonance readily facilitates the onset threshold required for initiation of temporal instability of the Faraday waves on the liquid layer and subsequent ejection of micro droplets at very low electrical drive power.

## 3. Temporal Instability of MHz Faraday Waves for Micro Droplet Ejection

### 3.1. Linear Theory

**Figure 5:**
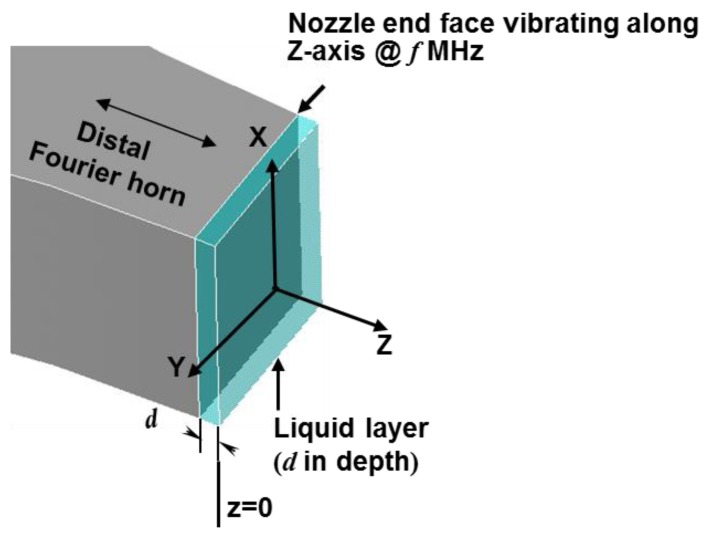
Rectangular coordinate system associated with liquid layer resting on the nozzle tip.

The enhanced longitudinal vibration amplitude (*h*) on the tip (end face of the distal Fourier horn) of the MFHUN, as treated in Section 2, exerts a periodic pressure on the planar liquid layer (thickness *d*) depicted in the inset of Figure 2b and Figure 5. Faraday waves are formed on the free surface of the liquid layer when the peak longitudinal vibration amplitude reaches the onset threshold or the critical amplitude (*h*_cr_). For convenience, the free surface of the liquid layer at rest is designated as the origin of the rectangular coordinate system. Thus, the amplitude of the Faraday wave $\text{​}\xi \;(\vec{x},t)$ represents the time-dependence displacement of the free liquid surface from *z* = 0, where $\vec{x}$ designates the two horizontal coordinates (*x*, *y*) parallel to the end face. The following theoretical treatment of the Faraday waves is based on mass-conservation and linearized Navier-Stokes equations for incompressible Newtonian liquids such as water, with density ρ, surface tension σ, and kinematic viscosity *υ* [13,14,16]. The three resulting equations are as follows:

(1) $$\nabla^{2}\Phi \left(\vec{x},\text{​ ​}z,\;t\right)=0,$$

(2) $$\partial_{t}\;\Phi \text{​ }\left(\vec{x},\text{​ ​}z,\;t\right)\;=\;-\Delta \;p(t)/\text{​}\rho +z\;g_{e}(t),$$

(3) $$(\partial_{t}-\upsilon \;\nabla^{2})\text{​ }\vec{u}\left(\vec{x},\text{​ ​}z,\;t\right)=0,$$

where Φ designates the velocity potential, Δ*p*(*t*) the pressure difference between the liquid side and the air side of the wavy liquid surface, *zg_e_*(*t*) is the potential energy in which *g_e_*(*t*) ≡ *h*(2π*f*)^2^cos(2π*ft*), and $\vec{u}\left(\vec{x},z,t\right)$ is the particle diffusive velocity that represents the rotational part of particle velocity $\vec{\upsilon }\left(\vec{x},z,t\right)\;$ . Again, *h* and *f* designate the longitudinal vibration displacement on the planar nozzle end face and the ultrasonic drive frequency, respectively. The kinematic condition on the liquid free surface is $\text{​}\upsilon_{z}\left(\vec{x},z,t\right){|}_{z=\text{​ }0}\;=\;\partial_{t}\;\xi \;(\vec{x},t)$ and on the interface with the nozzle end face is $\text{​}\upsilon_{z}\left(\vec{x},z,t\right){|}_{z\;=\;-\text{​ }d}\;=\;0$ , in which $\text{​}\upsilon_{z}\left(\vec{x},z,t\right)\;$ is the *z*-component of particle velocity $\vec{\upsilon }\left(\vec{x},z,t\right).\;$ The other boundary conditions to be satisfied on the liquid free surface are (i) the tangential stress vanishes and (ii) the normal stress satisfies the Laplace capillarity formula. Since the dimensions of the liquid layer on the nozzle end face are much larger than the wavelength (λ) of the standing Faraday waves (see Equation (9)) excited on its free surface, the liquid layer is assumed to be laterally unbounded. 

The four major steps involved in the theoretical treatment are as follows:

(1) Perform a Fourier transformation in the horizontal coordinates $\vec{x}$ for all the physical quantities and solve the Laplace Equation (1) for the velocity potential Φ*_k_* of the *k*-th-mode Faraday waves $\text{​}\xi_{k}\;(\vec{x},t)$ , 

(2) Obtain the governing equation for the *z*-component of particle diffusive velocity $\text{​}u_{z}\left(\vec{x},z,t\right)$ in terms of $\xi \left(\vec{x},t\right)$ ,

(3) Obtain the following equation for the temporal evolution of the amplitude ξ*_k_*(*t*) of the *k*-th mode Faraday waves $\text{​}\xi_{k}\;(\vec{x},t)$ :

(4) $$\partial_{t}^{2}\xi_{k}(t)+\;4\upsilon k^{2}\;\partial_{t}\xi_{k}(t)\;+(\omega_{k}^{2}+kg_{e}\left(t\right)\;)\xi_{\left(t\right)}^{k}\;=0$$

where the wave number is (*k*), the wavelength λ = 2π*/k*, $\omega_{k}^{2}\equiv \sigma \;k^{3}/\rho ,\;$ and the external acceleration *g_e_*(*t*) is equal to *h*(2π*f*)^2^cos(2π*ft*) in which *f* is the drive frequency. Note that the second term in Equation (4) represents the damping effect of viscosity, and 

**Figure 6:**
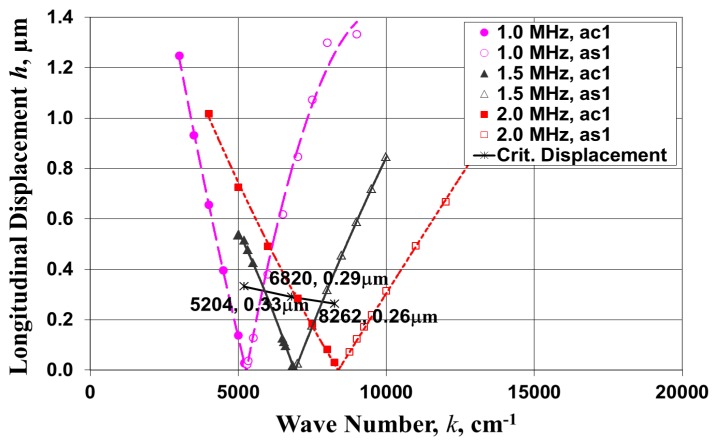
Stability charts and vibration amplitude thresholds (critical displacement) for Faraday wave instability in the most unstable region (bounded by the two characteristic curves designated as ac1 and as1 in the legend) at the drive frequencies of 1.0, 1.5, and 2.0 MHz.

(4) Finally, transform Equation (4) into a Mathieu Equation and solve it by means of Floquet’s Theorem for the standing Faraday waves with the wave number *k* (*k*-th mode) at a given external excitation amplitude *h* and drive frequency *f*. The resulting tongue-like plots of *h* vs. *k* are called stability (characteristic) charts [16,19]. Faraday waves grow unboundedly within the tongue-like regions of the charts. Only the first (most unstable) regions, bounded between the two characteristic curves ac_1_ and as_1_, represented by Mathieu functions, for the drive frequencies of 1.0, 1.5, and 2.0 MHz are calculated and shown in Figure 6 [16]. The corresponding fastest-growing Faraday wave amplitude ξ*_k_*(*t*) is thus given as follows [17]:

(5) $$\xi_{k}(t)=\;\xi_{0}e^{\pi \;k\text{​}f\;(h-h_{cr})\;t}\sin\left(2\pi \text{​}(f/2\text{​})\;t\;-\;\pi /4\right)\;,$$

where ξ_0_ designates the initial wave amplitude of the Faraday wave at half of the drive frequency (*f*/2) with the onset threshold or critical vibration amplitude (*h*_cr_) given as follows:

(6) $$h_{\mathrm{cr}}\;=2\upsilon k/(\pi \text{​}f\text{​})=2\upsilon \rho^{1/3}\text{​ }{\left(\pi \text{​​}\sigma \;\right)}^{-1/3}f^{-1/3}.$$

Equation (6) shows the specific dependence of the onset threshold on the drive frequency (*f*) and the liquid properties (ρ σ, and *υ*). It is important to emphasize that *h*_cr_ decreases with the drive frequency in accordance with *f*^−1/3^, and that the wave amplitude in Equation (5) grows exponentially in time when *h* > *h*_cr_; namely, when the longitudinal vibration amplitude exceeds the critical vibration amplitude. 

The characteristics charts of Figure 6 show that, as the drive frequency increases from 1.0 to 1.5 and to 2.0 MHz, the corresponding *h*_cr_ not only falls within the most unstable region but also decreases from 0.33 µm to 0.29 µm and to 0.26 µm, respectively. The corresponding Faraday wavelengths are 12.2, 9.2, and 7.6 µm, and the respective wave numbers are 5204, 6862, and 8262 cm^−1^ (see Equation (9)). While *h*_cr_ decreases with the drive frequency in accordance with *f*^−1/3^, the exponent π*kf*(*h–h*_cr_)*t* in the exponential factor of ξ*_k_*(*t*) increases with the drive frequency in accordance with *f*^4/3^. Thus, the temporal growth of the single-mode MHz Faraday wave excited at the fundamental subharmonic frequency (*f*/2) is very rapid once the nozzle tip (end face of the distal Fourier horn) excitation displacement *h* exceeds the critical value *h*_cr_.

### 3.2. Dynamics of Droplet Ejection and Droplet Diameter

As an example, take the Faraday waves at the 2.0 MHz drive frequency with the corresponding wavelength of 7.6 µm and *h*_cr_ of 0.26 µm in water and the periodic external acceleration *h*_cr_(2π*f*)^2^cos(2π*ft*) of 4.19 × 10^6^ *g*, in which *g* is the gravitational acceleration, the dynamics of droplet ejection are elucidated as follows:

When *h* exceeds the predicted *h*_cr_ of 0.26 µm by as little as 0.01 µm (*h* − 0.26 µm = 0.01 µm), the growth rate factor $e^{\pi kf\;(h-h_{\mathrm{cr}})\;t}\;$ of the Faraday wave amplitude in Equation (5) after a short time increment of 0.4 ms is 10^9^ times that with (*h* − *h*_cr_) as large as 100 µm at 200 Hz drive frequency with a corresponding low periodic acceleration of 16 *g* and a high *h*_cr_ of 100 µm [15] at the same time increment (0.4 ms). The amplitude growth rate factor at 2.0 MHz with (*h* − *h*_cr_) of 0.01 µm in a time increment of 0.4 ms is still greater (by 6%) than that at 200 Hz with (*h* − *h*_cr_) of 100 µm after a much longer time increment of 187 ms. Thus, the wave amplitude at 2 MHz drive frequency grows very rapidly, and, when it becomes too great to maintain wave stability, the Faraday waves break up to result in the ejection of droplets from the free surface of the liquid layer.

The ejected spherical droplets, with radius *a* and at the lowest oscillation mode frequency ω given by Equation (7) with *l* = 2, in which *l* designates the oscillation mode number [22], are dispersed in air and free from external acceleration (*g_e_* = 0). Thus, by setting the frequency of the droplet’s lowest oscillation mode equal to the nozzle drive frequency, the following theoretical droplet diameter (*D_p_* ≡ 2*a*) in terms of Faraday wavelength (λ) is obtained [16,22]:

(7) $$\omega^{2}\;=\;l\text{ ​}(l-1)\text{​ }(l+2)\text{​ }\sigma /\text{​}(\rho \text{​ }a^{3})$$

(8) $$D_{p}\;=\;2\;{\left(2/\pi^{2}\right)}^{1/3}{\left(\sigma /\rho \right)}^{1/3}f^{-2/3}\;=\;0.40\;\lambda$$

where

(9) $$\lambda \;=\;2\pi /\;k\text{ ​}=\;2\pi {\left({\rho \omega }_{k}^{2}/\sigma \right)}^{-1/3}={\left(8\pi \sigma /\rho \right)}^{1/3}f^{-2/3}.$$

Clearly, for a given liquid with known surface tension (σ) and density (ρ) to be atomized, the desired size of the droplets can be controlled by the drive frequency (*f*) of the MFHUN in accordance with *f^−2/3^*; the higher the nozzle drive frequency, the smaller the droplet diameter. 

## 4. Droplet Ejection Experiments and Verifications

All the droplet ejection (atomization) experiments were conducted using either the established bench-scale setup [18,19] or the battery-run pocket-size integrated droplet generator. Major components of the former are: (1) a MHz ultrasonic nozzle; (2) a tunable signal generator together with an amplifier to provide a MHz electrical drive to the ultrasonic nozzle; (3) a Syringe Pump Model #101 (KD Scientific Inc., Holliston, MA, USA) or a micro pump to provide a controlled flow rate of liquid to be atomized; (4) a charge-coupled device (CCD) camera to take pictures or movies of the droplet streams produced; and (5) a Malvern/Spraytec System Model #STP 5311 (Malvern Instruments Inc., Westborough, MA, USA) for measurement and analysis of the diameter and size distribution of the droplets generated. For the battery-run pocket-size integrated droplet generator the first three components are integrated in a module to be detailed in Section 5.

### 4.1. Critical Vibration Amplitude (h_cr_) for Droplet Ejection

The calculated *h*_cr_ values of 0.33 and 0.29 µm using Equation (6) for the formation of Faraday waves on water at 1.0 and 1.5 MHz drive frequencies, respectively, are slightly lower than the measured peak vibration displacements of 0.34 and 0.32 µm under droplet ejection using a laser Doppler vibrometer Model #PSV 400 (Polytech GmbH, Irvine, CA, USA) and are, thus, in excellent agreement with the theoretical prediction [16,22]. Thus, the very low electrical drive power requirement of subwatts for droplet ejection at MHz drive frequency presented in the following subsection is scientifically verified.

### 4.2. Droplet Diameter/Size Distribution and Electrical Drive Power

**Figure 7:**
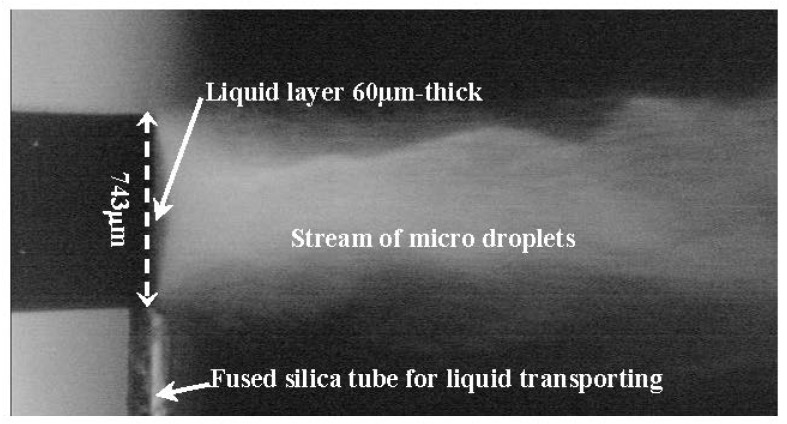
Droplet ejection from a 60 µm-water layer on the end face (743 µm × 800 µm) of a 2.0 MHz 4-Fourier horn nozzle at 200 μL/min output rate and 0.15 W electrical drive power.

As an example, Figure 7 shows a stream of micro droplets (aerosols) issuing from the tip (end face) of a 4-Fourier horn 2 MHz nozzle recently fabricated at an atomization frequency of 1.948 MHz, a throughput of 200 μL/min, and an electrical drive power of 0.15 W. Note that the liquid (water) was transported to the nozzle tip through a silica tube [23], and a 60 µm-water layer was maintained during atomization. 

**Figure 8:**
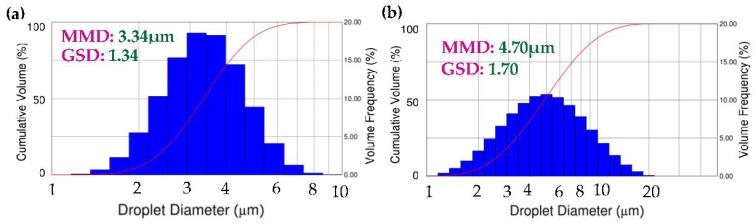
Diameter and size distribution of (**a**) water droplets produced by a 2 MHz 4-Fourier horn nozzle; and (**b**) aerosols produced by an advanced commercial ultrasonic nebulizer operating at 180 kHz (Omron NE-U22V).

The diameter and size distribution of the droplets (aerosols) produced by the 2.0 MHz nozzle were measured using a Malvern/Spraytec size analyzer (Model #STP 5311), and the data obtained is shown in Figure 8a. The measured droplet diameter (mass median diameter MMD) of 3.4 ± 0.2 µm is in very good agreement with the predicted value of 3.2 µm, based on Equation (8), and the corresponding GSD is 1.34. 

For easy comparison, Figure 8b was constructed in the same format as Figure 8a for the aerosols produced by an advanced commercial ultrasonic nebulizer at the drive frequency of 180 kHz with mass median aerodynamic diameter (MMAD) of 4.7 µm and a GSD of 1.70. Note that GSD stands for geometrical standard deviation. Droplets with a GSD of 1.0 are single sized and become more polydisperse with a larger GSD. Figure 8b clearly shows a much wider size distribution (GSD 1.70 vs. 1.34) and much smaller (<70% vs. ~100%) fine aerosol (particle) fractions (droplets smaller than 6 µm in diameter) than Figure 8a. Finally, at the typical throughput of 200 µL/min with 3.4 µm diameter droplets, which equals 1.5 × 10^8^ droplets per second, the required electrical drive power of 0.15 W corresponds to an energy requirement as low as 1 nano joule per droplet. Finally, a detailed analysis of the measured size distribution of the droplets produced showed no significant amounts of droplets with diameters that correspond to Faraday waves at frequencies higher than the fundamental (*f*/2) sub-harmonic frequency, such as 2(*f*/2) and 3(*f*/2) [13,19,22]. This finding suggested that the wave amplitude-related nonlinear effect was insignificant at the ultrasonic amplitudes involved in all actual droplet ejection experiments conducted thus far.

**Figure 9:**
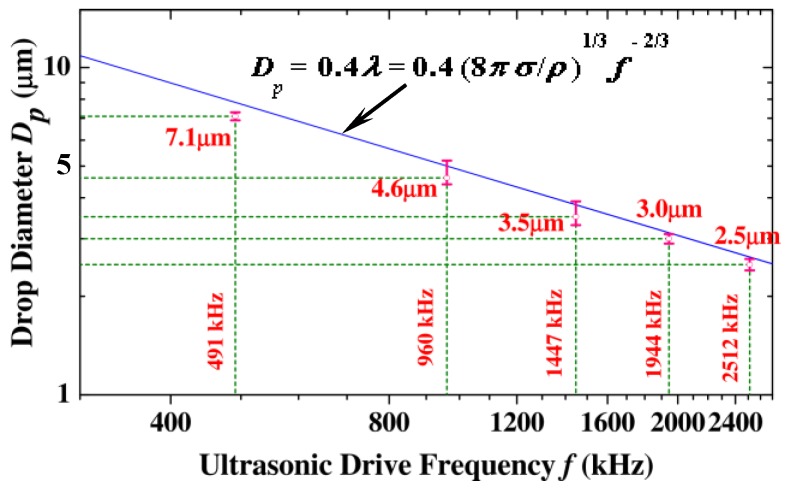
Comparison between the measured droplet diameters and the theoretical predictions vs. drive frequency.

During the past years of study, nozzles of increasing operation frequencies, namely, 0.5, 1.0, 1.4, 2.0, and 2.5 MHz, were fabricated and characterized, and the corresponding atomization performance in terms of droplet diameter, droplet-size distribution, ejection rate, and electrical drive power was measured. Figure 9 shows the very good agreement obtained between the measured droplet diameters and the theoretical diameters predicted by Equation (8) at the five nozzle drive frequencies [17,22]. 

In summary, all the experimental results obtained, including the critical vibration amplitude for droplet ejection, the controllable micrometer-size droplet diameter, narrow size distribution, and very low electrical drive power requirement, are in excellent agreement with the predictions of the linear theory on the temporal instability of MHz Faraday waves detailed in Section 3.

## 5. Battery-Run Pocket-Size Clogging-Free Integrated Ultrasonic Micro Droplet Generator

**Figure 10:**
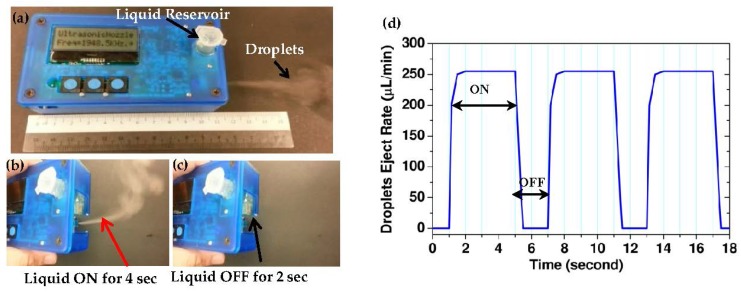
Battery-run pocket-size integrated ultrasonic micro droplet generator with electronically controlled ON/OFF capability: (**a**) the stand-alone unit; (**b**) nebulization turned ON; (**c**) nebulization turned OFF; and (**d**) nebulization ON/OFF time scales.

The centimeter-size nozzles with low electrical power requirements have enabled recent realization of battery-run pocketsize clogging-free integrated ultrasonic micro droplet generators. Figure 10a shows the 2 MHz unit with the pocketsize of 11.3 × 6.2 × 3.0 cm^3^. The integrated droplet generator module contains a single or a pair of 2 MHz 4-Fourier horn nozzles, an IC electronic driver, a cell-phone battery, a micro air pump, a liquid reservoir, and a liquid feed tube. Atomization (nebulization) rapidly reaches steady-state; i.e., the droplet ejection rate equals the liquid pumping rate (throughput) controlled by the pressure drop (the pressure differential between the liquid reservoir and the atmosphere) via the air pump. A typical throughput of 250 μL/min requires only sub-watt electrical drive power. The atomization ON/OFF times were varied electronically. Figure 10b,c depicts the ON/OFF throughput capability of the module, which is desirable in a variety of applications. The ON/OFF times of 4/2 s demonstrated in Figure 10d are compatible with adaptive therapy in inhalation drug delivery. As shown in the figure, it takes about ½-second for the liquid flow rate to decrease to zero and, thus, may lead to an error up to 12.5% in droplet ejection rate with the 4/2 s operation.

## 6. Applications

### 6.1. Imminent Application to Inhalation Drug Delivery

**Figure 11:**
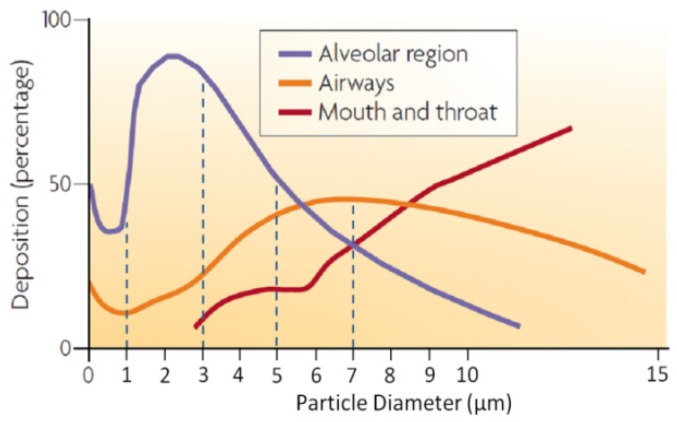
Particle deposition efficiency in human airways and lungs [25].

Inhalation is an important route for non-invasive drug delivery [24]. Drugs designed to treat pulmonary diseases or for systemic absorption through the lung require optimum particle (aerosol) size (2 to 6 µm), as shown in Figure 11, to target delivery [25]. Therefore, the control of aerosol size plays a critical role in the efficient and effective delivery of inhaled medications [26]. The minimization of upper airway deposition under different breathing conditions was correlated to Respirable Mass (RM), which is equal to Inhaled Mass (IM) multiplied by respirable fraction (RF), defined as fraction of diameters less than 2.5 µm [27]; the upper airway deposition decreased as the RM increased. As mentioned in the Introduction, even the advanced commercial inhalers/nebulizers using vibrating mesh technology still suffer from broad aerosol size (polydisperse) distributions and lack of size-control capability and are also plagued by the clogging of the orifices (diameter 2.5–5 µm) of the mesh used. In contrast, the MFHUNs do not require any mesh. Specifically, the liquid medicine flows from the outlet of the reservoir via a fused silica tube directly onto the vibrating nozzle end face and is atomized into aerosols. The preliminary version of a battery-run pocketsize nebulizer realized earlier using a single MFHUN had already been used successfully to aerosolize a variety of common pulmonary drugs (see Table 1), and its imminent application to inhalation drug delivery has been highlighted recently [17]. The controllability of particle (aerosol) size range (2.5 to 6 µm) and the much narrower size distribution demonstrated will improve the targeting of treatment within the respiratory tract and improve delivery efficiency. A recent in vitro experiment with Technetium (*T*_c_)-tagged saline solution has demonstrated higher delivery efficiency than the existing commercial nebulizers. Therefore, the MHz Faraday waves-based ultrasonic nebulizer would be a desirable device for inhalation delivery of expensive medicines such as gamma interferon [28]. 

**Figure 12:**
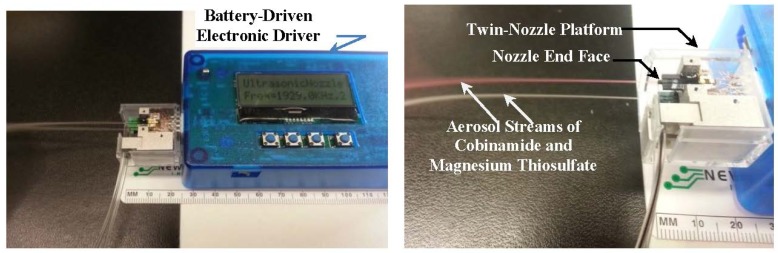
Battery-run pocket-size ultrasonic twin-nozzle nebulizer and twin-nozzle platform.

In addition, short treatment time is a critical requirement in acute situations such as massive cyanide poisoning [29,30]. Clearly, the treatment time can be shortened by increased aerosol output rate produced by an array of MFHUNs. Furthermore, nozzle arrays with individual nozzles operating at identical or different drive frequencies will provide the unique capability for the formation and subsequent mixing of aerosols of the same or different medicines at identical or different aerosol sizes. Note that such strategy is essential in order to avoid instability of mixed drug solutions prior to aerosolization. Figure 12 shows the battery-run pocket-size nebulizer with twin-nozzles which was constructed recently for simultaneous nebulization of cobinamide and magnesium thiosulfate antidotes for the detoxification of cyanide and sulfide poisoning [31]. The twin nozzles were designed with identical or separate specifications for the aerosol size (or drive frequency) and were driven by a pair of independent electronic drivers with controllable frequency and power, together with separate fused silica tubes for the transport of antidote solutions to the nozzle end faces. The twin-nozzle ultrasonic nebulizer has successfully demonstrated the capability for doubling the aerosol output of same antidote solution and the simultaneous aerosolization of different antidote solutions [31]. Specifically, for 100 mM cobinamide and 1 M magnesium thiosulfate antidote solutions, simultaneous and continuous aerosolization at the respective flow rates of 200 µL/min and 250 µL/min for 7 min. delivered 430 mg thiosulfate and 152 mg cobinamide, which would be sufficient antidote dosages for the effective detoxification of cyanide poisoning.

### 6.2. Other Potential Applications

The high-throughput micron-size monodisperse droplets produced by the MHz Faraday wave-based ultrasonic droplet generator reported here may find a wide range of attractive applications other than inhalation drug delivery [32]. Examples of such applications include the synthesis of nano particles via spray pyrolysis, sample injection in chemical analysis (e.g., mass spectroscopy), high-quality thin-film coating, 3-D photoresist coating of micro- and nano-structures in fabrication of nano-electronic and -photonic devices, acoustic deposition for matrix-assisted laser desorption/ionization (MALDI) sample preparation [33], fuel injection in micro combustors [34], delivery of lipid-based micro-encapsulated biological entities or genes [35,36], integration with microfluidic platforms for pharmaceutical preparations such as double emulsion [37,38], rapid heat removal in laser surgery, and the rapid administration of cosmetics.

## 7. Conclusions

The instability of Faraday waves at 0.5 to 2.5 MHz drive frequencies and silicon-based MHz multiple Fourier horn ultrasonic nozzles (MFHUNs) together have enabled the generation of narrowly sized droplets of controllable micron-size diameter (2.5–6.0 µm) at desirable throughput and subwatt electrical drive power. The resultant pocketsize battery-run clogging-free integrated droplet generator has established the potential for imminent application to inhalation (pulmonary) drug delivery.

## Figures and Tables

**Table 1:** Summary of drugs nebulized using 2 MHz multiple Fourier horn ultrasonic nozzles except specified.

Medicine	Medicine Concentration	Nebulizer Unit	Droplet MMD (µm)	Output Rate (µL/min)	Disease
Albuterol *	25 mg/mL	Bench-scale	4.5	150	Asthma
Humulin, U100 *	100 units/mL	Bench-scale	4.5	100	Diabetes
Cobinamide	100 mM	Pocket-size	3.7	150	Cyanide poisoning
Magnesium thiosulfate	1.0 M	Pocket-size	3.8	150	Sulfide poisoning
Interferon-γ	100 µg/0.5 mL	Pocket-size	2.9	100	Pulmonary fibrosis
Budesonide suspension	0.5 mg/2.0 mL	Pocket-size	3.1	350	Asthma

* Ultrasonic drive frequency at 1 MHz; all medicines listed in the table are aqueous solutions (except budesonide suspension) with viscosities similar to water (~1 cP) except 1.3 cP for Cobinamide. In an earlier study [23], liquids with viscosity up to 4.5 cP were successfully nebulized.
